# Prognosis-Related Nutritional Score for Cancer Patients (PRNS): a clinical nutritional score derived from a retrospective cohort study

**DOI:** 10.1186/s12967-022-03696-x

**Published:** 2022-10-20

**Authors:** Bingdong Zhang, Yuerui Li, Yongbing Chen

**Affiliations:** 1 Department of Gastrointestinal Surgery/Department of Clinical Nutrition, Beijing Shijitan Hospital, Capital Medical University, Beijing, China; 2 Beijing International Science and Technology Cooperation Base for Cancer Metabolism and Nutrition, Beijing, 100038 China; 3 Key Laboratory of Cancer FSMP for State Market Regulation, Beijing, China; 4 Department of Cardiology, The Second Medical Center and National Clinical Research Center for Geriatric Diseases, Chinese PLA General Hospital, Beijing, China; 5 Beijing Key Laboratory of Chronic Heart Failure Precision Medicine, Beijing, China

**Keywords:** Nutrition screening and assessment, Patient-generated subjective global assessment (PG-SGA), Investigation on Nutritional Status and its Clinical Outcomes of Common Cancers (INSCOC), 30-item European Organization for Research and Treatment of Cancer Core Quality of Life Questionnaire (EORTC QLQ-C30), Transparent Reporting of a Multivariable Prediction Model for Individual Prognosis or Diagnosis (TRIPOD), Cancer, Prognostic value

## Abstract

**Background:**

Nutritional assessment and quality of life (QOL) have become important indices for therapeutic efficacy in patients with malignancies. We aim to develop and validate an easy-to-use questionnaire with prognostic value to assess nutritional status in hospitalized cancer patients.

**Methods:**

A comprehensive survey focused on patient-generated subjective global assessment (PG-SGA) and 30-item European Organization for Research and Treatment of Cancer Core Quality of Life Questionnaire (EORTC QLQ-C30 Chinese version) was performed in a cohort of 22,776 patients derived from the INSCOC study. Among them, 1948 patients were followed for 3 years after admission. An observational, retrospective, cross-sectional cohort study was conducted in accordance with TRIPOD statement. Breiman's random forest model was applied to calculate variable importance (VIMP) for items in PG-SGA and EORTC QLQ-C30 (Chinese version) for nutritional recommendation. Cox regression model was employed to construct Prognosis-Related Nutritional Score for Cancer Patients (PRNS). Kaplan–Meier Survival curve, ROC and DCA were calculated to evaluate prognostic value of nutritional status categorized by PRNS, and compared with PG-SGA.

**Results:**

Nutritional status was classified into 4 levels by PRNS scores: well nourished (≤ 4.5 points), mild malnourished (5–7.5 points), moderate malnourished (8–14.5 points), and severe malnourished (≥ 15 points). Significant median overall survival differences were found among nutritional status groups stratified by the PRNS (all Ps < 0.05). Compared with PG-SGA, PRNS had better prognostic value for survival stratified by nutritional status. The external, internal validity, test–retest reliability and rater reliability were satisfactory.

**Conclusions:**

We systematically developed and validated PRNS as a nutrition screening tool for cancer patients. Compared with PG-SGA, PRNS has better prognostic value and simpler operation.

**Trial registration:**

Investigation on Nutrition Status and its Clinical Outcome of Common Cancers, ChiCTR1800020329. Registered 24 December 2018—Retrospectively registered, http://www.chictr.org.cn/showproj.aspx?proj=31813

**Supplementary Information:**

The online version contains supplementary material available at 10.1186/s12967-022-03696-x.

## Introduction

Due to various etiologic factors, 20–50% of patients are malnourished or at high risk of malnutrition upon hospital admission [1–3]. The incidence of malnutrition in cancer patients has been reported as between 39 and 87% [4]. Malnutrition leads to decreased muscle activity, weakened immune function, worsened postoperative complications, prolonged hospitalization, increased psychological and economic burden, as well as seriously undermined quality of life [5–7]. Early identification of patients at risk of malnutrition or who are malnourished will be crucial for timely and adequate nutritional support. Preoperative nutritional evaluation and treatment is necessary for most cancer patients. The purpose of nutritional assessment is to predict clinical outcome related to nutritional status and treatment. A number of nutrition assessment tools have been developed [8–14]. However, there is no gold standard for evaluation of nutritional status currently [15].

The patient-generated-subjective global assessment (PG-SGA) was adapted from the Subject Global Assessment (SGA) and developed specifically for patients with cancer [16–20]. PG-SGA is the most widely used tool for evaluating nutritional status for cancer patients, exhibiting better sensitivity, specificity, as well as positive and negative predictive values compared with other tools. The PG-SGA scoring system has been accepted by the Oncology Nutrition Dietetic Practice Group of the American Dietetic Association as the standard for nutrition assessment in patients with cancer. PG-SGA questionnaire has been designed to include components of medical history, which could be completed by a patient using a check box format. Then, physical examination is performed by a health professional, e.g., physician, nurse or dietitian. It usually takes about 15 min to complete a PG-SGA questionnaire [21]. In clinical diagnosis and therapy, PG-SGA has been found to be time-consuming. Another nutrition assessment tool, abPG-SGA, uses patient-generated component (Additional file 1: Box 1–4) as a simplified version, without modification and statistical analysis for item selection [12, 14]. The abPG-SGA usually serves as a nutrition screening tool rather than an inpatient assessment tool. Therefore, there is an unmet need to develop a simpler nutrition assessment tool for cancer patients, which may predict clinical outcomes.

In this study, a comprehensive survey included PG-SGA and Chinese version of 30-item European Organization for Research and Treatment of Cancer Core Quality of Life Questionnaire (EORTC QLQ-C30) was performed in a cohort of 22,776 patients from the Nutrition Status and Clinical Outcome of Common Cancers (INSCOC) study. An observational, retrospective, cross-sectional cohort study was conducted in accordance with the Transparent Reporting of a Multivariable Prediction Model for Individual Prognosis or Diagnosis (TRIPOD) statement. Subsequently, we developed and validated “PRNS” as a nutrition assessment tool for cancer patients.

## Materials and methods

### Population

This cohort was a part of the INSCOC study (Registration number: ChiCTR1800020329), and performed from May 2013 to April 2021 in 72 tertiary hospitals in China. The INSCOC study is a nationwide cross-sectional survey on association between nutritional status and clinical outcome in patients with malignant tumors conducted by the Chinese Society of Nutritional Oncology (CSNO) [22]. Participants were older than 18 years with pathologically diagnosed malignant tumor, conscious (without communication disorders in Chinese), as well as willing to participate in this study and to sign an informed consent form. Exclusion criteria were as follows: (1) AIDS or organ transplantation; (2) in a critical condition and difficult to be assessed; (3) refuse to or cannot cooperate with a questionnaire. All admitted patients were interviewed by professionals to complete a formatted questionnaire including the PG-SGA and Chinese version of EORTC QLQ-C30 (V3.0). The questionnaire was administered within 48 h after admission by physicians and or specialist nutrition nurses who had received standardized training. The main workflow of data processing was described in Additional file 1: Fig. S1. Until April 5, 2021, 41,587 patients were initially included in this study. Subjects with incomplete questionnaires/missing variables (n = 18,811) were excluded from statistical analysis. The current study included 22,776 patients. Among them, 1,948 were followed up for more than 3 years after admission (Additional file 1: Fig. S1). The median follow-up time was 14.90 months (range: 0.03–85.03 months), during which there were 1381 deaths. This study was approved by the Ethics Committee of each participating hospital and complied with the Declaration of Helsinki.

### Data collection and analysis

All measurements were performed by trained medical professionals. Nutritional status was evaluated by PG-SGA, and QOL was assessed by EORTC QLQ-C30 (Chinese version).

### Assessment method

The PG-SGA was adapted from SGA and developed specifically for hospitalized patients with cancer. PG-SGA includes detailed history and physical assessment parameters in seven domains [16] as follows: Additional file 1: Box 1, weight and worksheet 1, scoring weight loss (0–5 points); Additional file 1: Box 2, food intake (0–4 points); Additional file 1: Box 3, symptoms (0–23 points); Additional file 1: Box 4, activities and functions (0–3 points); Additive score of the Additional file 1: Box 1–4 is recorded in Additional file 1: Box A; worksheet 2 (recorded in Additional file 1: Box B), disease (0–6 points); worksheet 3 (recorded in Additional file 1: Box C), metabolic demand (0–9 points); and worksheet 4 (recorded in Additional file 1: Box D), physical examination (0–3 points). Based on PG-SGA scores, nutritional status could be categorized into four levels [23]. 0–1 point was considered as well-nourished, whereas 2–3 points as mildly malnourished, 4–8 points as moderately malnourished, and ≥ 9 points as severely malnourished (Additional file 1: Table S4).

The EORTCQLQ-C30 systematically evaluates QOL in cancer patients. The Chinese version of EORTC QLQ-C30 (V3.0) has been proven to be valid, reliable, and clinically relevant [24–26]. It involves 30 topics summarized as three categories to define symptoms (fatigue, nausea and vomiting, pain), five categories to qualify functions (physical, role, emotional, cognitive, and social function), six single measurement subjects (difficulty in breathing, insomnia, loss of appetite, constipation, diarrhea, economic difficulties), and one score to assess overall QOL (Supplementary Table 5).

### Statistical analysis

Statistical analysis was performed with R package (version 3.3.2) via RStudio interface (version 1.0.136). The R package “glmnet” (version 4.0–2) was applied to perform LASSO Cox analysis on the basis of overall survival (OS). The risk score plot for Cox regression was plotted with R package “ggrisk” (version 1.2). Random survival forests algorithm implemented in the R randomForestSRC was applied to rank survival-related items by relative importance. The OS was defined as the length of time from the date of assessment to the date of death or the date of the last follow-up for censored patients. The OS was calculated using Kaplan–Meier plot and log-rank test. A p < 0.05 was considered statistically significant.

## Results

### Characteristics of patients

Characteristics of patients recruited in this study without follow-up were described in Table 1. PG-SGA classified 18.9% of patients (n = 3939) as well nourished. 81.1% (n = 16,889) of the patients indicated necessity of nutritional management: 20.1% (n = 4181) were defined as mild malnutrition (2–3 points); 30.8% (n = 6412) as moderate malnutrition (4–8 points); whereas 30.2% (n = 6296) as severe malnutrition (≥ 9 points). The distribution of primary tumor location in this study was similar to that released by WHO in 2020 (https://www.iarc.who.int/).

**Table 1:** Patient characteristics

Characteristics	Male	Female	Total
n (%)	11783(56.6)	9045(43.4)	20828
Age (years) (Mean ± SD)	58.8 ± 11.9	54.8 ± 12.1	57.0 ± 12.2
Nutrition status(PG‐SGA score), n (%)			
Well nourished(0–1)	1887(16.0)	2052(22.7)	3939(18.9)
Mild malnutrition(2–3)	2369(20.1)	1812(20.0)	4181(20.1)
Moderate malnutrition(4–8)	3751(31.8)	2661(29.4)	6412(30.8)
Severe malnutrition(≥ 9)	3776(32.0)	2520(27.9)	6296(30.2)
Primary tumor location, n (%)			
Lung cancer	3120(26.5)	1506(16.7)	4626(22.2)
Colorectal cancer	2174(18.5)	1559(17.2)	3733(17.9)
Gastric cancer	1862(15.8)	797(8.8)	2659(12.8)
Breast cancer	21(0.2)	1895(21.0)	1916(9.2)
Esophageal cancer	1305(11.1)	257(2.8)	1562(7.5)
Nasopharyngeal carcinoma	877(7.4)	309(3.4)	1186(5.7)
Leukemia	402(3.4)	350(3.9)	752(3.6)
Cervical cancer	0(0.0)	751(8.3)	751(3.6)
Malignant lymphoma	411(3.5)	301(3.3)	712(3.4)
Liver cancer	408(3.5)	133(1.5)	541(2.6)
Ovarian cancer	0(0.0)	431(4.8)	431(2.1)
Pancreatic cancer	189(1.6)	134(1.5)	323(1.6)
Brain cancer	125(1.1)	94(1.0)	219(1.1)
Endometrial cancer	0(0.0)	160(1.8)	160(0.8)
Prostate cancer	193(1.6)	0(0.0)	193(0.9)
Bladder cancer	126(1.1)	34(0.4)	160(0.8)
Biliary cancer	54(0.5)	44(0.5)	98(0.5)
GIST	12(0.1)	12(0.1)	24(0.1)
Other cancer	504(4.3)	278(3.1)	782(3.8)

### PG-SGA could predict prognosis in cancer patients

**Fig. 1:**
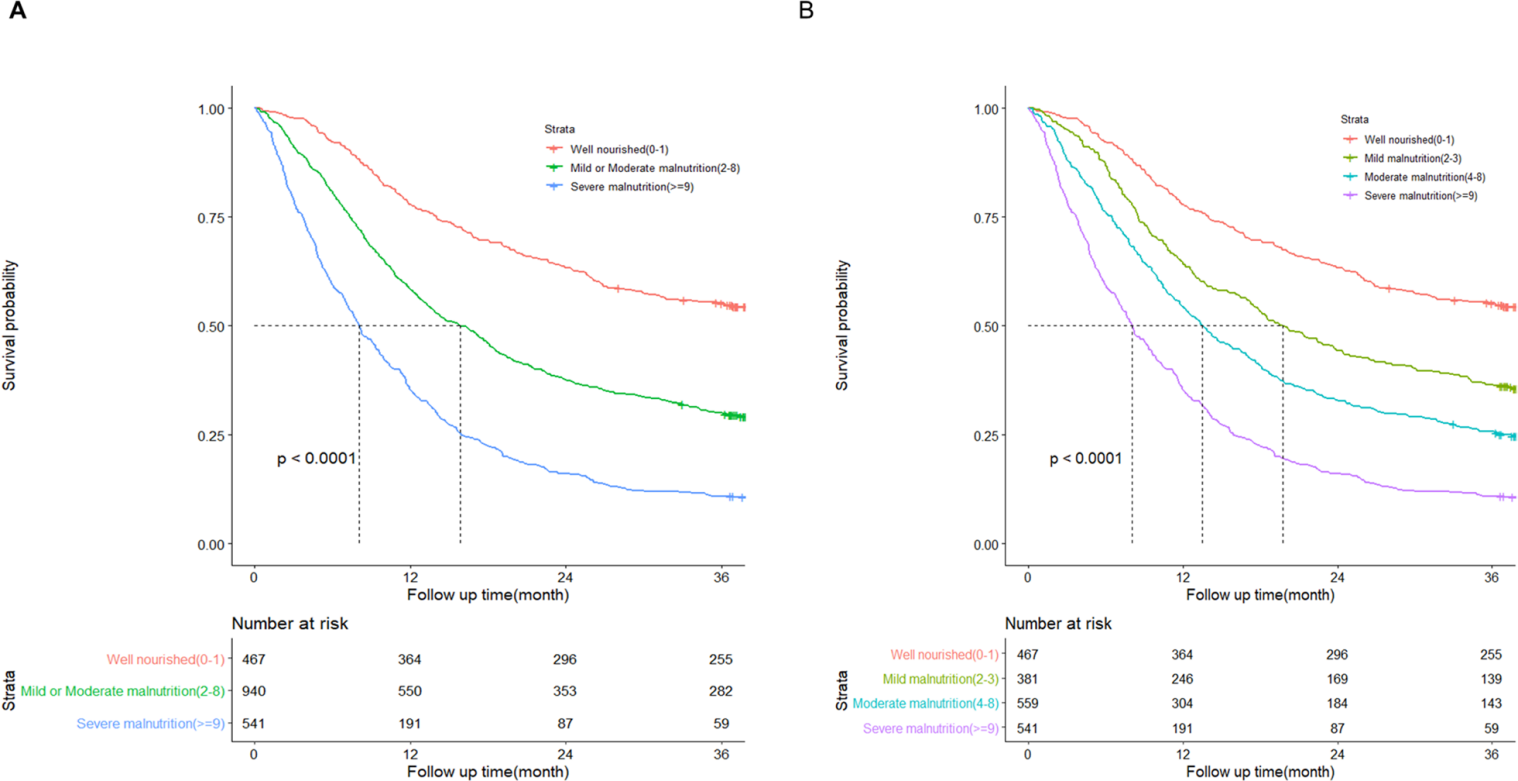
Kaplan–Meier curves for overall survival time of cancer patients in different nutrition status diagnosed by PG-SGA. **A** Kaplan–Meier survival analysis stratified by PG-SGA. Well nourished (0–1) = Stage A; Mild or Moderate malnutrition (2–8) = Stage B; Severe malnutrition (> = 9) = Stage C. **B** Kaplan–Meier survival analysis stratified by PG-SGA. Well nourished (0–1); Mild malnutrition (2–3); Moderate malnutrition (4–8); Severe malnutrition (> = 9). Overall survival between different nutrition status groups were analyzed and compared by Kaplan–Meier analysis and log Rank test. *PG-SGA* Patient-Generated Subject Global Assessment

PG-SGA could predict prognosis in cancer patients (Fig. 1). The relationship between the nutritional status evaluated by the PG-SGA and patient overall survival (OS) was examined using Kaplan–Meier methods. OS was defined as the time from the date of PG-SGA evaluation to the date of death from any cause. Kaplan–Meier curves indicated significant differences among different categories of PG-SGA classification grouped by global assessment (Fig. 1A) or nutritional triage recommendations (Fig. 1B). Based on survival curves, patients with severe malnutrition had the shortest survival time.

### Important factors affecting nutritional status and prognosis of cancer patients

**Fig. 2:**
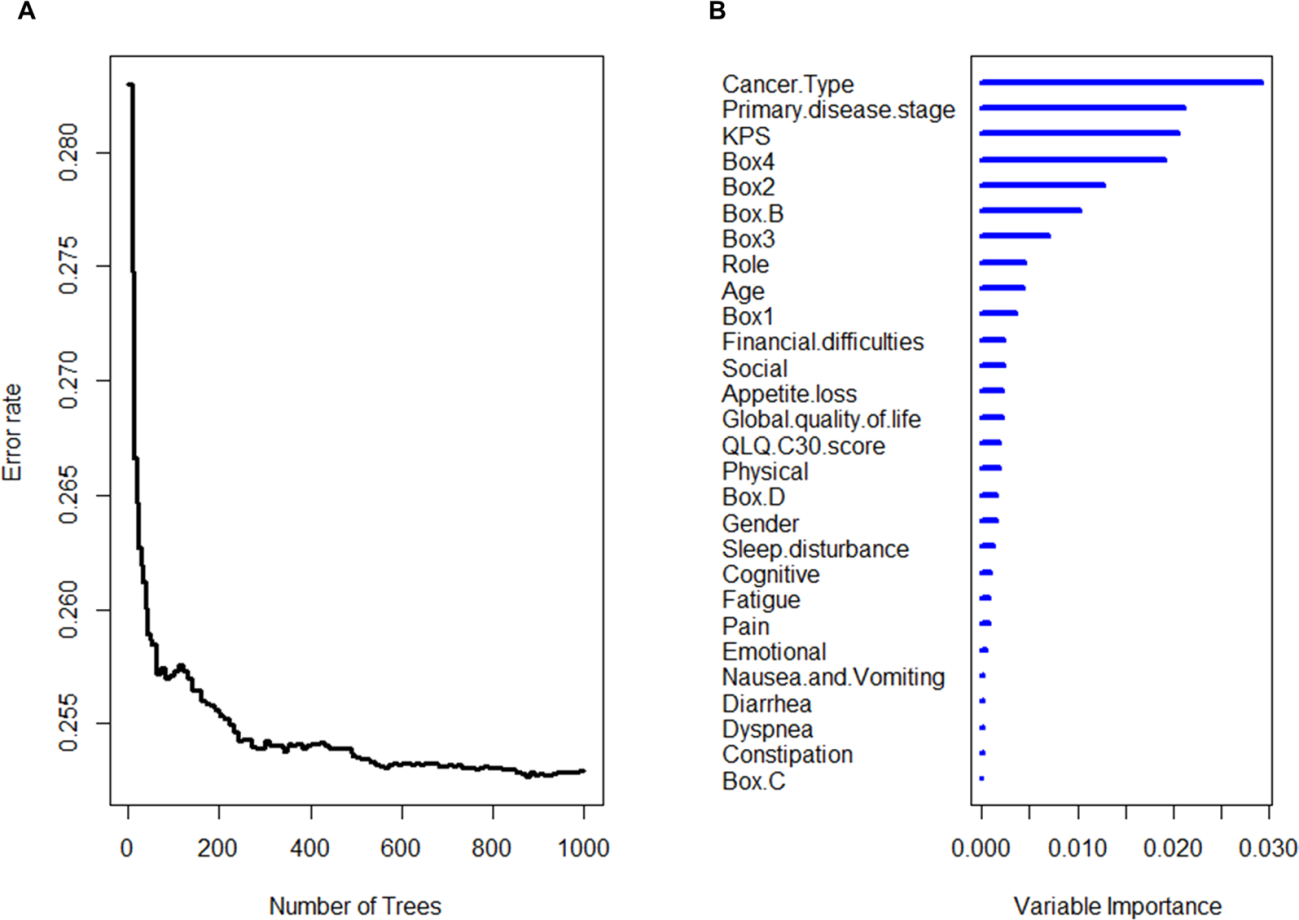
Variable importance of the candidate items in INSCOC study. **A** The relationship between the error rate and the number of classification trees. **B** The importance of the candidate items in INSCOC study for individual prognostic evaluation. *KPS* Karnofsky performance status; QLQ-C30 functioning scales: Physical, Role, Cognitive, Emotional, Social, Global quality of life; QLQ-C30 Symptom scales: Fatigue, Nausea and Vomiting, Pain, Dyspnea, Sleep disturbance, Appetite loss, Constipation; Primary disease stage: TNM stage; PG-SGA: Additional file 1: Box1–4 are completed by the patient, Additional file 1: Box B, C, D are completed by doctor, nurse, therapist

Our study aims to develop a simple nutritional assessment that can predict the prognosis of cancer patients. The INSCOC study provides us with a large number of candidate items. To evaluate individual prognostic value of each candidate item in INSCOC study, we utilized random survival forests algorithm (Ntree = 1,000, default parameters of Hemant Ishwaran algorithm), and set age, gender, cancer type, primary disease stage, Karnofsky performance status (KPS), scales in QLQ-C30 and boxes in PG-SGA as variables in this model (Fig. 2). All items were ranked by relative importance after processing random survival forests algorithm with R software (Fig. 2B). Cancer type and primary disease stage were the most important prognostic factors, followed by KPS. However, cancer type and stage could not be changed by intervention, so could not become candidates for the new scale. Activities and function (Record in Additional file 1: Box 4) were important components of nutritional status. Additional file 1: Box 4 in PG-SGA had good prognostic value similar to KPS. Therefore, activities and function were parts of the new scale.

Many factors can affect the prognosis of cancer patients. Factors indicating nutritional status from these candidates should be identified. All items in PG-SGA and QLQ-C30 (Additional file 1: Tables S1, S2) were ranked by relative importance after processing random forests algorithm via R software for predicting nutritional status (PG-SGA classification grouped by global assessment). Not all items in PG-SGA indicated nutritional status of patients (Additional file 1: Fig. S2). Thus, final new scale only retained a small number of candidates in PG-SGA.

### Selection of candidate items for assessment

**Fig. 3:**
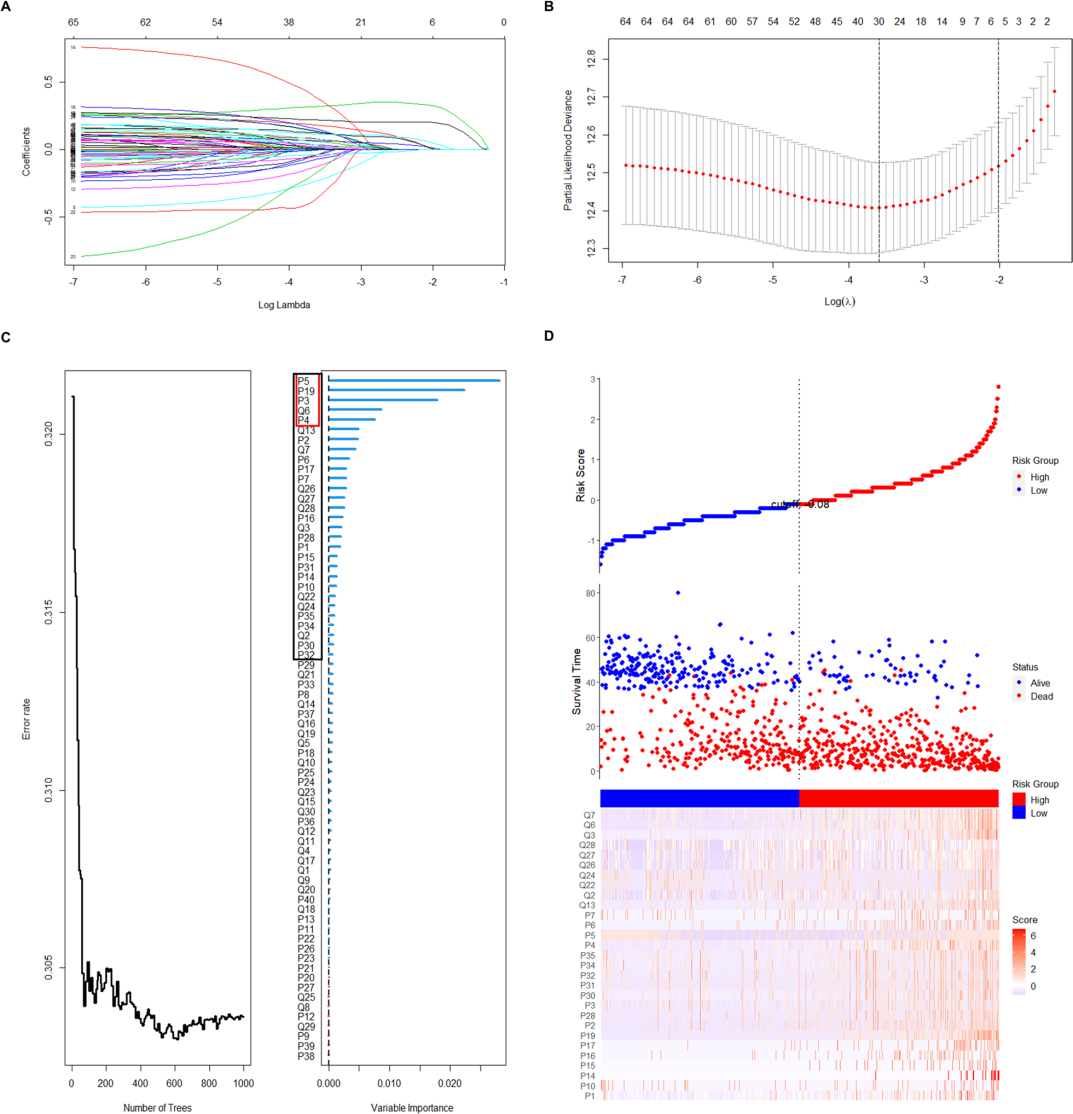
Selection of candidate items. **A** The predictive factors were determined by cable regression method. **B** Adjusting the penalty coefficient in the LASSO model using cross validation and minimum criteria. The vertical black line represents the optimal lambda (i.e., the model provides the best fit with the data). **C** The importance of the candidate items in PG-SGA and QLQ-C30 for individual prognostic evaluation. The relationship between the error rate and the number of classification trees (left). The variables are ranked in terms of importance (right, red rectangle: minimal variables; black rectangle: optimal variables). **D** The curve of risk score, survival status of the patients and heat map of optimal variables scores were shown

To determine an optimal number of items for assessment, 70 items were incorporated in the LASSO Cox regression model, including P1-P40 and Q1-Q30. According to λ value, the number of items should range between 5 and 29 (Fig. 3A, B). In order to select the most weighted features, we utilized random survival forests algorithm (Ntree = 1000, default parameters of Hemant Ishwaran algorithm), and set 70 items as variables in this model. These features were ranked by relative importance after processing random survival forests algorithm via R software (Fig. 3C). A total of 29 features were retained as candidate items. To explored potential effects of these 29 features on prognosis, scatter plots corresponding to survival time in different patients were generated (Fig. 3D). The risk score constructed from 29 features could predict the prognosis of patients. However, Q22, Q24, Q26, Q27 and Q28 were excluded due to no relationship with nutritional status. Many previous studies had confirmed that physical exam in PG-SGA was difficult to operate [27]. Among these 29 features, P28, P30, P31, P32, P34 and P35 belonged to physical exam with a similar scoring pattern (Fig. 3D). For the convenience of operation, P31 was reserved as the only item for physical exam. Q13 and P6 evaluated the same content, so Q13 was deleted. The final candidate items included Q2, Q3, Q6, Q7, P1, P2, P3, P4, P5, P6, P7, P10, P14, P15, P16, P17, P19 and P31.

### Establishment of PRNS

**Fig. 4:**
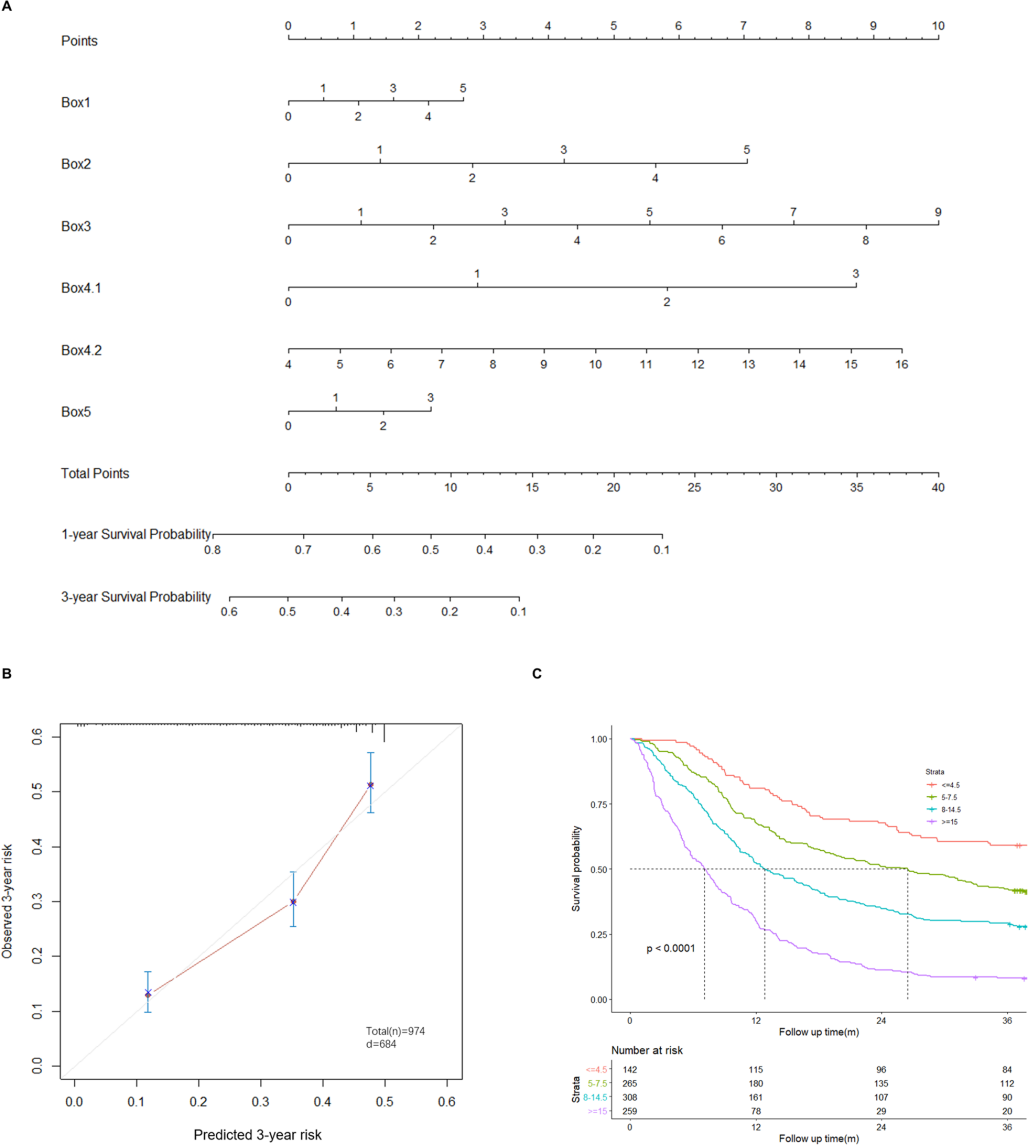
Establishment of PRNS. **A** Nomogram of patients OS combining the 18 reserved items. **B** The 3-year overall survival (OS) nomogram calibration curves. **C** In the training cohort, Kaplan–Meier curves for overall survival time of cancer patients in different nutrition status diagnosed by nomogram

After selection, 18 reserved items were subjected to nomogram construction with “rms” R package in the training dataset (Fig. 4A). The calibration plots (3 years) were used to evaluate the prognostic accuracy of nomogram. Based on calibration plots, observed vs. predicted proportion of 3-year OS exhibited good concordance (Fig. 4B). According to relative importance of items in nomogram, scoring was formulated as follows: total score = 0.5 × Box1 + 1.5 × Box2 + Box3 + 3 × Box4.1 + Box4.2 + Box5. Then, all patients were scored and sorted from small to large. According to the proportion of nutritional status as listed in Table 1, cut-off value of nutritional status was obtained and construction of a new scale, PRNS was completed (Additional file 1: Table S3). Based on cut-off values, all patients were divided into four groups. Kaplan–Meier survival curves indicated significant differences among these groups (Fig. 4C).

### Validation of PRNS

**Fig. 5:**
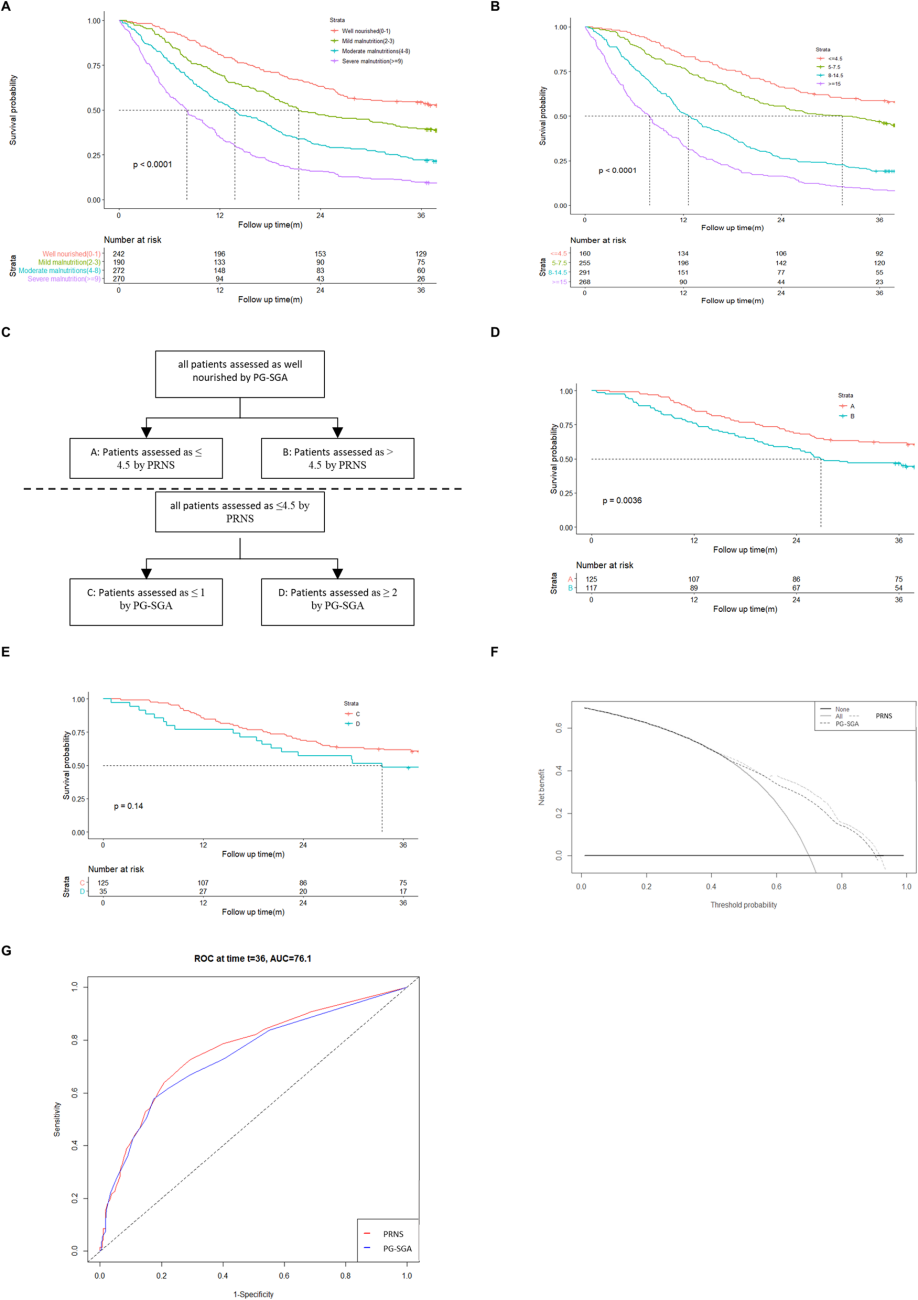
Validation of PRNS. **A** Kaplan–Meier survival analysis stratified by PG-SGA. Well nourished (0–1); Mild malnutrition (2–3); Moderate malnutrition (4–8); Severe malnutrition (> = 9). **B** Kaplan–Meier survival analysis stratified by PRNS. Well nourished (< = 4.5); Mild malnutrition (5–7.5); Moderate malnutrition (8–14.5); Severe malnutrition (> = 15). **C** Patients assessed as well nourished by PG-SGA in the testing cohort were regrouped by PRNS: A (≤ 4.5) and B (> 4.5); Patients assessed as well nourished by PRNS in the testing cohort were regrouped by PG-SGA: C (≤ 1) and D (≥ 2). **D** Kaplan–Meier survival analysis of A and B. **E** Kaplan–Meier survival analysis of C and D. **F** Decision curve analysis (DCA) of the clinical utility between PG-SGA and PRNS regarding the overall survival (OS) in the testing cohort. **G** AUC of PRNS and PG-SGA in the testing cohort (t = 36 months)

In the validation dataset, Kaplan–Meier survival curves indicated significant differences among different categories of PG-SGA classification (Fig. 5A). Similarly, the four groups stratified by PRNS had significantly different outcomes. Kaplan–Meier curves suggested higher scores significantly associated with worse survival (Fig. 5B). The most important function of nutritional assessment scale for cancer patients is to identify potential risk of malnutrition and to guide interventional treatment for survival benefit. In the validation dataset, all patients assessed as well nourished by PG-SGA were re stratified according to PRNS (panels A and B in Fig. 5C). Kaplan–Meier survival analysis indicated a significant difference between A and B (Fig. 5D, p = 0.0036). Similarly, patients assessed as ≤ 4.5 by PRNS were re stratified according to PG-SGA (panels C and D in Fig. 5C). Kaplan–Meier survival analysis indicated no significant difference between C and D (Fig. 5E, p = 0.14). The DCA results were shown in Fig. 5F. In the validation dataset, PRNS had a better net benefit than PG-SGA.

We then calculated continuous NRI for clinical risk model for death at 3 years. had a continuous NRI of 33.4% (95% CI: 19.92–46.89%; P = 0.0000). To evaluate the discriminatory performance of PRNS and PG-SGA, receiver operating characteristic (ROC) analysis was performed (Fig. 5G) predicting the technical outcome: PRNS AUC 0.761, 95% CI: 0.728 –0.792; PG-SGA AUC 0.738, 95% CI: 0.706–0.770; comparison of AUCs: p = 0.024.

### Clinical application of PRNS

**Fig. 6:**
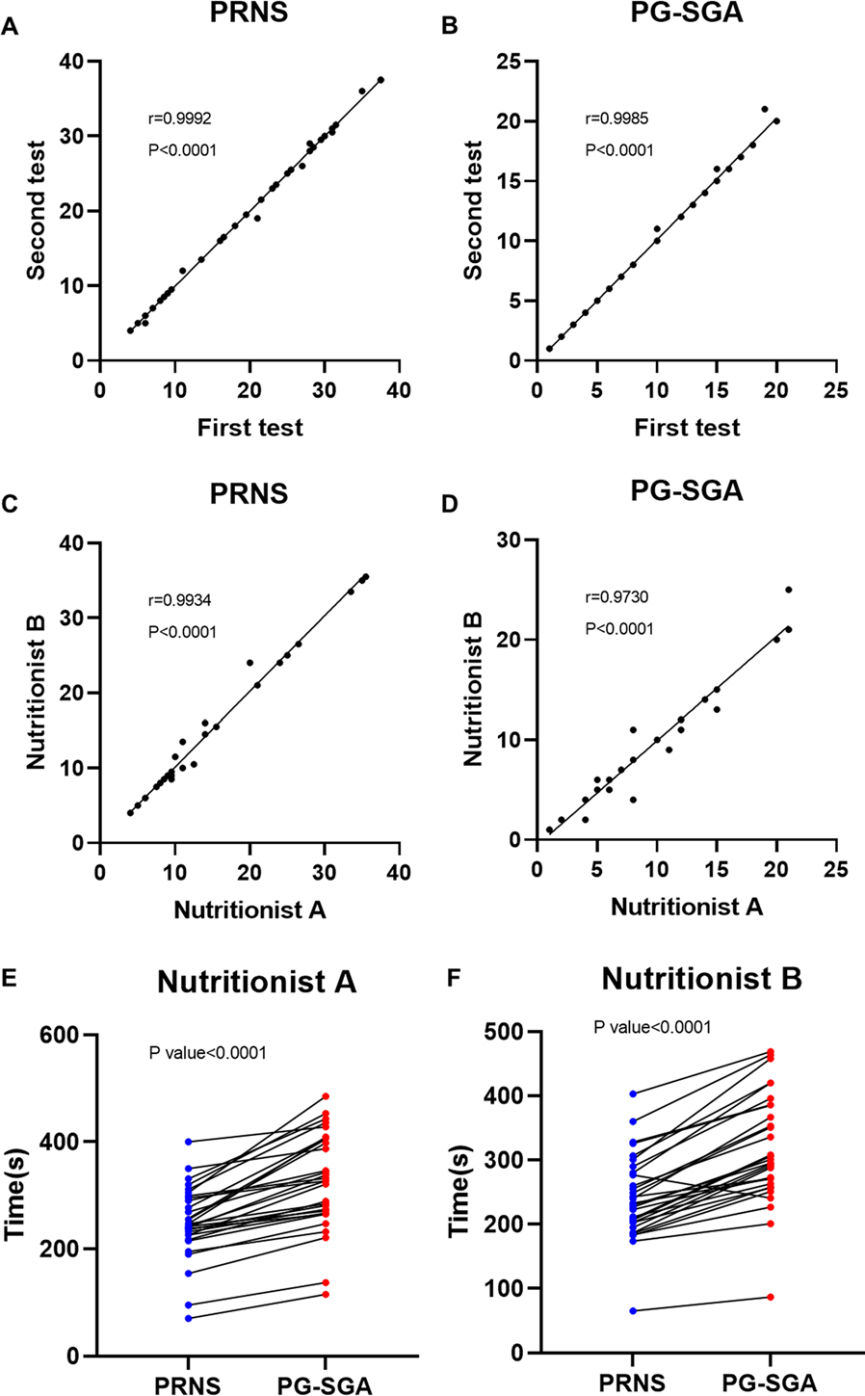
Clinical application of PRNS. **A** Scatterplots illustrating the associations between two tests of PRNS at 24-h intervals. **B** Scatterplots illustrating the associations between two tests of PG-SGA at 24-h intervals. **C** Scatterplots illustrating the associations between two tests of PRNS by two raters at an interval of 24 h. **D** Scatterplots illustrating the associations between two tests of PG-SGA by two raters at an interval of 24 h. **E** The operation time of PRNS and PG-SGA on the same patient by nutritionist A. **F** The operation time of PRNS and PG-SGA on the same patient by nutritionist B

In order to explore application value of PRNS, we randomly recruited 49 cancer patients to examine test–retest reliability. There was no significant difference between the two tests at an interval of 24 h (Fig. 6A, Pearson r = 0.9992). Similar results were obtained for PG-SGA (Fig. 6B Pearson r = 0.9985). In addition, we recruited 30 cancer patients to examine rater reliability. There was no significant difference between the two raters at an interval of 24 h (Fig. 6C Pearson r = 0.9934). Similar results were obtained for PG-SGA (Fig. 6D Pearson r = 0.9730). There was no significant difference in test–retest reliability or rater reliability between the two scales. However, PRNS was simpler, its stability was better. The operation time required for PRNS was also significantly shorter than that of PG-SGA (Fig. 6E and F).

## Discussion

In this study, we developed and validated PRNS, an updated/improved format of PG-SGA. This is an attempt to systematically design and validate a nutritional assessment tool for cancer patients based on INSCOC study in data-driven mode. The simplicity and better prognostication value of PRNS for survival make it an optimal clinical nutritional status assessment tool.

With a rapid increase in incidence of cancer [28], more attention has been paid on patient’s nutrition status. Nutritional care plays a central role in appropriate management of cancer patients. The INSCOC aims to determine the prevalence of malnutrition in cancer patients in China as well as its relationship with QOL and clinical outcomes. In INSCOC, PG-SGA was applied to assess cancer patient’s nutritional status routinely. PG-SGA works well for assessment of nutrition status for cancer patients after 8 years of application. Recent studies reported that 30–70% of patients at risk of malnutrition received nutritional assessment and half of them received an appropriate intervention. The difficulty in operation of PG-SGA should be kept in mind. It is a challenge to implement routine nutritional assessment in busy and resource-constrained wards. Therefore, it is necessary to develop a simple and reliable scale, which can predict the prognosis of cancer patients. In such a context, abPG-SGA and other easier tools were built up [18, 29–31]. Most of these tools were generated by simply deleting parts of PG-SGA without rigorous examination. Thus, there is an unmet need to systematically develop a simple scale as a nutrition assessment tool with better prognostication value to guide clinical treatment.

At present, PRNS is an improved format of PG-SGA derived from INSCOC based on data-driven mode. The INSCOC has provided sufficient samples for research. The distribution of primary tumor location in this study is similar to that released by the WHO in 2020 (https://www.iarc.who.int/). A relatively large number of cases could be representative for different cancer types and to determine optimal cutoffs. Here, we have conducted an observational, retrospective, cross-sectional cohort study in accordance with TRIPOD statement [32]. Breiman's random forest model was employed to calculate variable importance (VIMP) for items in PG-SGA and EORTC QLQ-C30 (Chinese version). Currently, there is no gold standard to estimate cancer patients’ nutritional status. The most important function of nutritional assessment scale for cancer patients is to identify potential risk of malnutrition and to guide interventional treatment for survival benefit. Now, PRNS not only evaluates nutritional status, but also focuses on prediction of patients' prognosis. PRNS indicates that malnutrition contributes to poor prognosis. During clinical application, operation time required for PRNS is significantly shorter than that of PG-SGA, although physicians and/or specialist nutrition nurses are very familiar with PG-SGA. Thus, PRNS may have the potential to replace PG-SGA in evaluating nutritional status for hospitalized cancer patients.

This study has several limitations. All patients included in this research were from tertiary hospitals in China. Therefore, these findings may not be necessarily generalizable to other populations. Thus, extensive international collaboration and validation are required to improve and confirm predictive/applicative value of PRNS. In this study, cut-off value of PRNS was obtained according to the proportion of population-based nutritional status grouped by PG-SGA. More accurate cut-off value needs to be adjusted in a national wide cohort for long-term follow-up. In order to generalize our results, larger prospective cohort studies are guaranteed.

## Conclusion

We have systematically developed and validated PRNS as a nutrition evaluation tool for cancer patients. Compared with PG-SGA, validity of PRNS has been strengthened dependent on better prognostication value of nutritional status for survival. More importantly, PRNS is easier for clinical use.

## Supplementary Information

**Additional file 1: Figure S1**. Flow chart. PG-SGA, Patient-Generated Subject Global Assessment. INSCOC, Investigation on Nutrition Status and its Clinical Outcome of Common Cancers. QLQ-C30, The 30-item Research and Treatment of Cancer Core Quality of Life Questionnaire. **Figure S2**. The importance of items in PG-SGA and QLQ-C30 for nutritional status evaluation. **Table S1**. All items in PG-SGA. **Table S2**. All items in EROTC QLQ-C30. **Table S3**. Prognosis-Related Nutritional Score for Cancer Patients (PRNS). **Table S4**. Patient-Generated Subjective Global Assessment (PG-SGA). **Table S5**. EROTC QLQ-C30.

## Data Availability

Data described in the article will be made available upon request pending application and approval.

## Abbreviations

PG-SGA: Patient-generated subjective global assessment
INSCOC: Investigation on Nutritional Status and its Clinical Outcomes of Common Cancers
EORTC QLQ-C30: 30-Item European Organization for Research and Treatment of Cancer Core Quality of Life Questionnaire
TRIPOD: Transparent Reporting of a Multivariable Prediction Model for Individual Prognosis or Diagnosis;
PRNS: Prognosis-Related Nutritional Score for Cancer Patients
QOL: Quality of life
SGA: The Subject Global Assessment
CSNO: The Chinese Society of Nutritional Oncology
OS: Overall survival
ROC: Receiver operating characteristic
