# Supplementary material for: Prognosis-Related Nutritional Score for Cancer Patients (PRNS): a clinical nutritional score derived from a retrospective cohort study
Source: J Transl Med. 2022 Oct 20;20:477. doi: 10.1186/s12967-022-03696-x (PMC9583551; doi:10.1186/s12967-022-03696-x)
Supplement: Supplementary file 1 — Additional file 1: Figure S1. Flow chart. PG-SGA, Patient-Generated Subject Global Assessment. INSCOC, Investigation on Nutrition Status and its Clinical Outcome of Common Cancers. QLQ-C30, The 30-item Research and Treatment of Cancer Core Quality of Life Questionnaire. Figure S2. The importance of items in PG-SGA and QLQ-C30 for nutritional status evaluation. Table S1. All items in PG-SGA. Table S2. All items in EROTC QLQ-C30. Table S3. Prognosis-Related Nutritional Score for Cancer Patients (PRNS). Table S4. Patient-Generated Subjective Global Assessment (PG-SGA). Table S5. EROTC QLQ-C30. [file 12967_2022_3696_MOESM1_ESM.docx]

Supplementary Figure 1


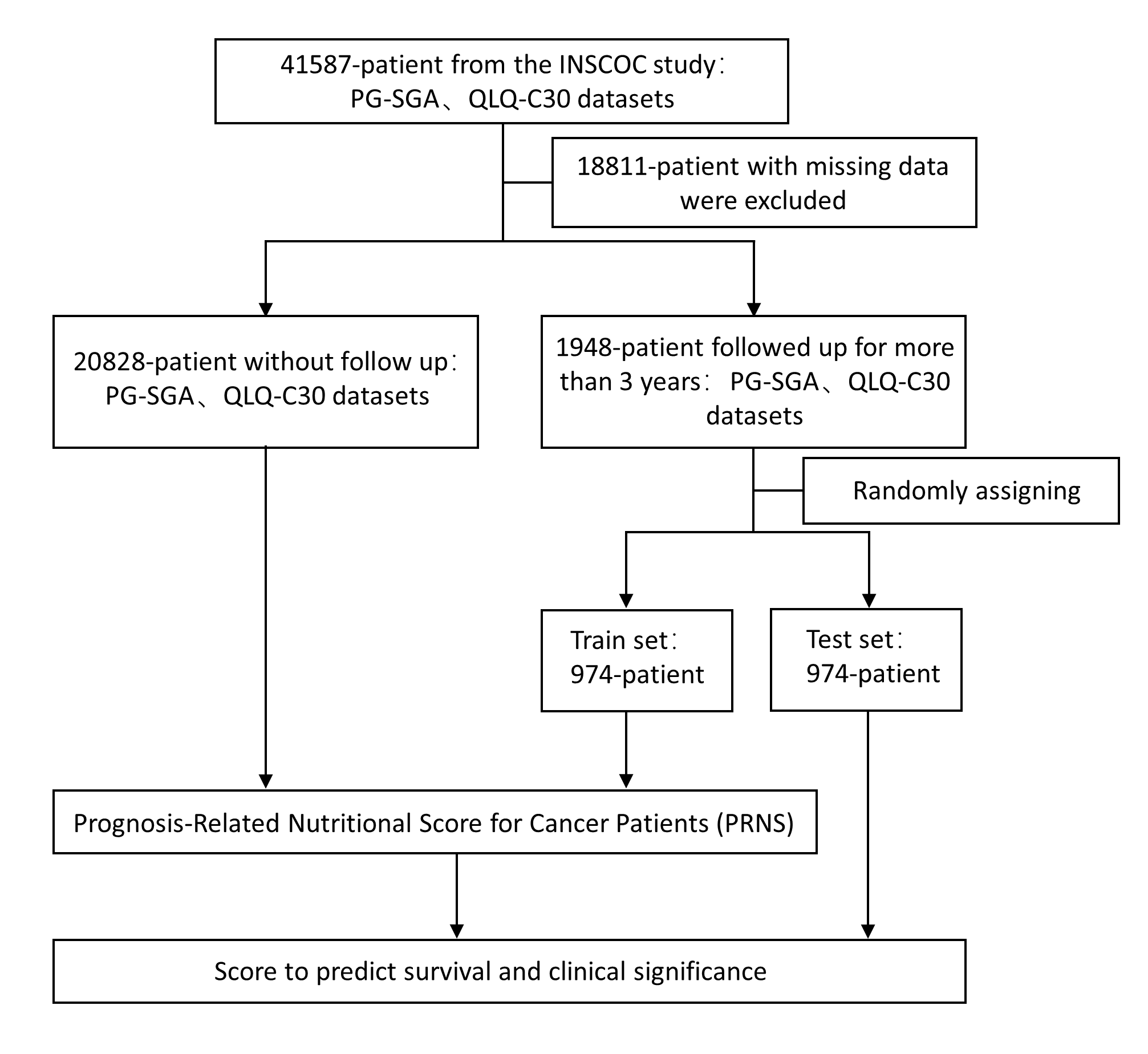
 **Supplementary Figure 1.** Flow chart. PG-SGA, Patient-Generated Subject Global Assessment. INSCOC, Investigation on Nutrition Status and its Clinical Outcome of Common Cancers. QLQ-C30, The 30-item Research and Treatment of Cancer Core Quality of Life Questionnaire.

Supplementary Figure 2


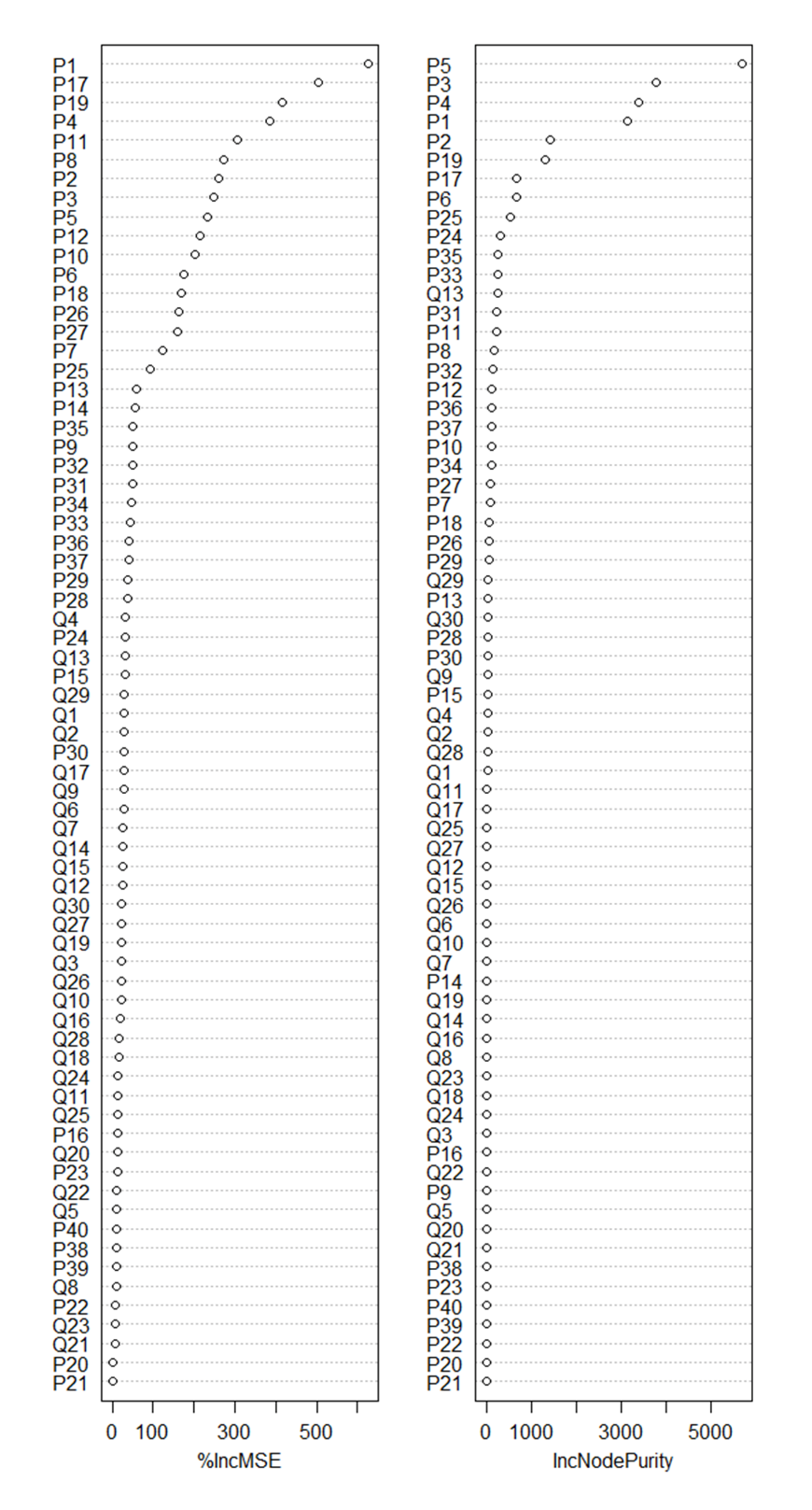


**Supplementary Figure 2.** The importance of items in PG-SGA and QLQ-C30 for nutritional status evaluation.

**Supplementary Table 1.** **All items in PG-SGA.**

| **Identifier** | **Item** |
| --- | --- |
| P1 | Weight loss |
| P2 | Weight loss within 2 weeks |
| P3 | Food intake during the past month |
| P4 | Food intake: I am now taking |
| P5 | No problems eating |
| P6 | No appetite just did not feel like eating |
| P7 | Nausea |
| P8 | Vomiting |
| P9 | Mouth sores |
| P10 | Constipation |
| P11 | Diarrhea |
| P12 | Dry mouth |
| P13 | Things taste funny or have no taste |
| P14 | Smell bother me |
| P15 | Problem swallowing |
| P16 | Feel full quickly |
| P17 | Pain |
| P18 | Others |
| P19 | Activities and function over the past month I would generally rate my activity as. |
| P20 | AIDS |
| P21 | Pulmonary or cardiac cachexia |
| P22 | Presence of decubitus open wound or fistula |
| P23 | Presence of trauma |
| P24 | Age greater than 65 years |
| P25 | Disease score |
| P26 | Fever |
| P27 | Glucocorticoids dose |
| P28 | Orbital fat pads |
| P29 | Triceps skin fold |
| P30 | Fat overlying lower ribs |
| P31 | Temporalis muscle |
| P32 | Pectoralis deltoids |
| P33 | Deltoids |
| P34 | Interosseous muscles |
| P35 | Latissimus dorsi trapezius deltoids |
| P36 | Quadriceps |
| P37 | Gastrocnemius |
| P38 | Ankle edema |
| P39 | Sacral edema |
| P40 | Ascites |

**Supplementary Table 2.** **All items in EROTC QLQ-C30.**

| **Identifier** | **Item** |
| --- | --- |
| Q1 | Do you have any trouble doing strenuous activities, like carrying a heavy shopping bag or a suitcase? |
| Q2 | Do you have ang trouble taking a long walk? |
| Q3 | Do you have ang trouble taking a short walk outside of the house? |
| Q4 | Do you have to stay in a bed or a chair for most of the day? |
| Q5 | Do you need help with eating, dressing, washing yourself or using the toilet? |
| Q6 | Are you limited in any way in doing either you work or doing household jobs? |
| Q7 | Are you limited in your hobbies or leisure activities? |
| Q8 | Were you short of breath? |
| Q9 | Have you had pain? |
| Q10 | Did you need to rest? |
| Q11 | Have you had trouble sleeping? |
| Q12 | Have you felt weak? |
| Q13 | Have you lacked appetite? |
| Q14 | Have you felt nauseated? |
| Q15 | Have you vomited? |
| Q16 | Have you been constipated? |
| Q17 | Have you had diarrhea? |
| Q18 | Were you tired? |
| Q19 | Did pain interfere with your daily activities? |
| Q20 | Have you had difficulty in concentrating on things, like reading a newspaper or watching television? |
| Q21 | Did you feel tense? |
| Q22 | Did you worry? |
| Q23 | Did you feel irritable? |
| Q24 | Did you feel depressed? |
| Q25 | Have you had difficulty remembering things? |
| Q26 | Has your physical condition or medical treatment interfered with your family life? |
| Q27 | Has your physical condition or medical treatment interfered with your social activities? |
| Q28 | Has your physical condition or medical treatment caused you financial difficulties? |
| Q29 | How would you rate your overall physical condition during the past week? |
| Q30 | How would you rate your overall quality of life during the past week? |

**Supplementary Table 3. Prognosis-Related Nutritional Score for Cancer Patients (PRNS)**

| Box 1. Weight | Wt loss in 1 month | | | Points | Wt loss in 6 months | | Box 1. Score |
| --- | --- | --- | --- | --- | --- | --- | --- |
| 1.1 In summary of my current and recent weight:  I currently weigh about _______ kg  I am about _________ cm tall  One month ago, I weighed about _________ kg  Six months ago, I weighed about _________ kg  1.2 During the past two weeks my weight has:  decreased (1) not changed (0) increased (0) | 10% or greater  5-9.9%  3-4.9%  2-2.9%  0-1.9% | | | 4  3  2  1  0 | 20% or greater  10 -19.9%  6 - 9.9%  2 - 5.9%  0 - 1.9% | |  |
|  | Use 1 month weight data if available. Use 6 month data only if there is no 1 month weight data. Add one extra point if patient has lost weight during the past 2 weeks. | | | | | |  |
| Box 2. Food Intake: As compared to my normal intake, I would rate my food intake during the past month as: | | | | | | | Box 2. Score |
| unchanged (0)  more than usual (0)  less than usual (1)  If less than usual, I am now taking:  normal food but less than normal amount (1)  little solid food (2)  only liquids or nutritional supplements (3)  very little of anything (4)  only tube feedings or only nutrition by vein (0) | | | | | | | Use the highest score checked, no matter how many checked |
| Box 3. Symptoms: I have had the following problems that have kept me from eating enough during the past two weeks (check all that apply): | | | | | | | Box 3. Score |
| no problems eating (0)  no appetite, just did not feel like eating (3)  nausea (1) constipation (1) smells bother me (1) feel full quickly (1) problems swallowing (2) pain; where? (3) _ | | | | | | | Add all points for Box 3 total score |
| Box 4. Activities and Function: Over the past month, I would generally rate my activity as: | | | | | | | Box 4. Score |
| 4.1  normal with no limitations (0)  not my normal self, but able to be up and about with fairly normal activities (1)  not feeling up to most things, but in bed or chair less than half the day (2)  able to do little activity and spend most of the day in bed or chair (3)  pretty much bedridden, rarely out of bed (3) | | | | | | | Add 4.1 points*3 and 4.2 points for Box 4 total score. |
| 4.2 | Not at All | A Little | Quite a Bit | | | Very Much |  |
| with trouble taking a long walk (1 2 3 4)  with trouble taking a short walk outside of the house (1 2 3 4)  limited in any way in doing either you work or doing household jobs (1 2 3 4)  limited in your hobbies or leisure activities (1 2 3 4) | | | | | | |  |
| Box 5. Physical |  | | | | | | Box 5. Score |
| Temples(temporalis. muscle) | Definition of categories: 0 = no deficit, 1+ = mild deficit, 2+=moderate deficit, 3+ = severe deficit. | | | | | | Use the highest score checked |
| Total PG-SGA Score  (Total numerical score of 0.5*Box1+1.5*Box2+Box3+Box4+Box5 above)  (See triage recommendations below) | | | | | | | |
| Nutritional Triage Recommendation: Additive score is used to define specific nutritional interventions including patient & family education, symptom management including pharmacologic intervention, and appropriate nutrient intervention(food, nutritional supplements, enteral, or parenteral triage). First line nutrition intervention includes optimal symptom management.  <=4.5 No intervention required at this time. Re-assessment on routine and regular basis during treatment.  5-7.5 Patient & family education by dietitian, nurse, or other clinician with pharmacologic intervention as indicated by symptom survey (Box3) and laboratory values as appropriate.  8-14.5 Requires intervention by dietitian, in conjunction with nurse or physician as indicated by symptoms survey (Box3).  >=15 Indicates a critical need for improved symptom management and/or nutrient intervention options. | | | | | | | |

**Supplementary Table 4. Patient-Generated Subjective Global Assessment (PG-SGA)**

| **Scored Patient-Generated Subjective Global Assessment (PG-SGA)**  © FD Ottery, 2001 | Patient ID Information |
| --- | --- |
| **History (Boxes 1-4 are designed to be completed by the patient)** |  |
| 1. **Weight** (See Worksheet 1)   In summary of my current and recent weight:  I currently weigh about _______ kg  I am about _________ cm tall  One month ago, I weighed about ________kg  Six months ago, I weighed about ________kg  During the past two weeks my weight has:  □decreased (1) □not changed (0) □increased (0)  Box 1 | 2. **Food Intake**: As compared to my normal intake, I would rate my food intake during the past month as:  □ unchanged (0)  □ more than usual (0)  □less than usual (1)  I am now taking:  □normal food but less than normal amount (1)  □little solid food (2)  □only liquids (3)  □only nutritional supplements (3)  □very little of anything (4)  □only tube feeding or only nutrition by vein (0)  Box 2 |
| 3. **Symptoms:** I have had the following problems that have kept me from eating enough during the past two weeks (check all that apply):  □no problems eating (0)  □no appetite, just did not feel like eating (3)  □nausea (1) □vomiting (3)  □constipation (1) □diarrhea  □mouth sores □dry mouth  □things taste funny or have no taste □smells bother me (1) □feel full quickly (1) □problems swallowing (2) □pain; where? (3) _______  □other**(1)________________  **Examples: depression, money, or dental problem  Box 3 | 4. **Activities and Function:** Over the past month, I would generally rate my activity as:  □normal with no limitations (0)  □not my normal self, but able to be up and about with fairly normal activities (1)  □not feeling up to most things, but in bed or chair less than half the day (2)  □able to do little activity and spend most of the day in bed or chair (3)  □pretty much bedridden, rarely out of bed (3)  Box 4 |
|  | Additive Score of the Boxes 1-4 A |

| **The remainder of this form will be completed by your doctor, nurse, or therapist. Thank you.** | |
| --- | --- |
| 5.**Disease and its relation to nutritional requirements** (See Worksheet 2)  All relevant diagnoses (specify) ______________________________________  Primary disease stage (circle if known or appropriate) Ⅰ Ⅱ Ⅲ Ⅳ Other________  Age________ **Numerical score from Worksheet 2** **B**  **6.Metabolic Demand** (See Worksheet 3)  **Numerical score from Worksheet 3** C  7.**Physical** (See Worksheet 4)  **Numerical score from Worksheet 4** D | |
| Global Assessment (See Worksheet 5)  □Well-nourished or anabolic (SGA-A)  □Moderate or suspected malnutrition (SGA-B)  □Severely malnourished (SGA-C) | **Total PG-SGA score**  **(Total numerical sore of A+B+C+D above)**  (See triage recommendations below) |

| **Nutritional Triage Recommendations:** Additive score is used to define specific nutritional interventions including patient & family education, symptom management including pharmacologic intervention, and appropriate nutrient intervention (food, nutritional supplements, enteral, or parenteral triage). First line nutrition intervention includes optimal symptom management.  0-1 No intervention required at this time. Re-assessment on routine and regular basis during treatment.  2-3 Patient & family education by dietitian, nurse, or other clinician with pharmacologic intervention as indicated by symptom survey (Box 3) and laboratory values as appropriate.  4-8 Requires intervention by dietitian, in conjunction with nurse or physician as indicated by symptom survey (Box 3).  ≥9 Indicates a critical need for improved symptom management and/or nutrient intervention options. |
| --- |

| **Worksheets for PG-SGA Scoring**  © FD Ottery, 2001  Boxes 1-4 of the PG-SGA are designed to be completed by the patient. The PG-SGA numerical score is determined using  1) the parenthetical points noted in boxes 1-4 and 2) the worksheets below for items not marked with parenthetical points. Scores for boxes 1 and 3 are additive within each box and scores for boxes 2 and 4 are based on the highest scored item checked off by the patient. | |
| --- | --- |
| **Worksheet 1 Scoring Weight (Wt) Loss**  To determine score, use I month weight data if available. Use 6 month data only if there is no I month weight data. Use points below to score weight change and add one extra point if patient has lost weight during the past 2 weeks. Enter total point score in Box I of the PG-SGA.  Wt loss in 1 month Points Wt loss in 6 months  10% or greater 4 20% or greater  5 - 0 9% 3 10 - 19.9%  3 - 4.9% 2 6 - 9.9%  2 - 2.9% 1 2 - 5.9%  0 - 1% 0 0 - 1.9%  **Score for Worksheet 1**  Record in Box 1 | **Worksheet 2 Scoring Criteria for Condition**  Score is derived by adding 1 point for each of the conditions listed below that pertain to the patient.  **Category Points**  Cancer 1  AIDS 1  Pulmonary or cardiac cachexia 1  Presence of decubitus, open wound, or fistula 1  Presence of trauma 1  Age greater than 65 years 1  **Score for Worksheet 2**  Record in Box B |
| **Worksheet 3 Scoring Metabolic Stress**  Score for metabolic stress is determined by a number of variables known to increase protein & calorie needs. The score is additive so that a patient who has a fever of > 102 degrees (3 points) and is on 10 mg of prednisone chronically (2 points) would have an additive score for this section of 5 points.  Stress none (0) low (1) moderate (2) high (3)  Fever no fever >99 and <101 ≥101 and <102 ≥102  Fever duration no fever <72 hrs 72 hrs > 72 hrs  Steroids no steroids low dose moderate dose high dose steroids  (<10mg prednisone (≥10 and <30mg (≥30mg prednisone  equivalents/day) prednisone equivalents/day)  equivalents/day)  **Score for Worksheet 3**  Record in Box C | |
| **Worksheet 4 Physical Examination**  Physical exam includes a subjective evaluation of 3 aspects of body composition: fat, muscle, & fluid status. Since this is subjective, each aspect of the exam is rated for degree of deficit. Muscle deficit impacts point score more than fat deficit. Definition of categories: 0 = no deficit, 1+ = mild deficit, 2+ =moderate deficit, 3+ = severe deficit. Rating of deficit in these categories are not additive but are used to clinically assess the degree of deficit (or presence of excess fluid). | |
| **Fat Stores:**  Orbital fat pads 0 1+ 2+ 3+  Triceps skin fold 0 1+ 2+ 3+  Fat overlying lower ribs 0 1+ 2+ 3+  **Global fat deficit rating** 0 1+ 2+ 3+  **Muscle Status:**  Temples (temporalis muscle) 0 1+ 2+ 3+  Clavicles (pectoralis & deltoids) 0 1+ 2+ 3+  Shoulders (deltoids) 0 1+ 2+ 3+  Interosseous muscles 0 1+ 2+ 3+  Scapula  (latissimus dorsi, trapezius, deltoids) 0 1+ 2+ 3+  Thigh (quadriceps) 0 1+ 2+ 3+  Calf (gastrocnemius) 0 1+ 2+ 3+  **Global muscle status rating** 0 1+ 2+ 3+ | **Fluid Status:**  ankle edema 0 1+ 2+ 3+  sacral edema 0 1+ 2+ 3+  ascites 0 1+ 2+ 3+  **Global fat deficit rating** 0 1+ 2+ 3+ |
|  | Point score for the physical exam is determined by the overall subjective rating of total body deficit.  No deficit score = 0 points  Mild deficit score = 1 point  Moderate deficit score = 2 points  Severe deficit score = 3 points  **Score for Worksheet 4 =**  **Record in Box D** |
| **Worksheet 5 PG-SGA Global Assessment Categories**  **Stage A Stage B Stage C**  Category Well-nourished Moderately malnourished Severely malnourished  or suspected malnutrition  Weight No wt loss OR ~5% wt loss within 1 mouth >5% wt loss in 1month  Recent non-fluid wt gain (or 10% in 6mouths) OR ( >10% in 6months) OR  No wt stabilization or wt gain No wt stabilization or wt gain  (i.e., continued wt loss) (i.e., continued wt loss)  Nutrient Intake No deficit OR Definite decrease in intake Severe deficit in intake  Significant recent improvement  Nutrition Impact Symptoms None OR Presence of nutrition impact Presence of nutrition impact  Significant recent improvement symptoms (Box 3 of PG-SGA) symptoms (Box3 of PG-SGA)  allowing adequate intake  Functioning No deficit OR Moderate functional deficit OR Severe functional deficit OR  Significant recent improvement Recent deterioration recent significant deterioration  Physical Exam No deficit OR Evidence of mild to moderate Obvious signs of malnutrition  Chronic deficit but with recent loss of SQ fat&/or muscle mass （e.g., severe loss of SQ tissues,  Clinical improvement &/or muscle tone on palpation possible edema)  **Global PG-SGA rating (A, B, or C) =** | |

**Supplementary Table 5. EROTC QLQ-C30**

| Please fill in your initials:  Your birthdate (Day, Month, Year):  Today's date (Day, Month, Year): | | | | | |
| --- | --- | --- | --- | --- | --- |
|  |  | Not at all | A little | Quite a bit | Very much |
| 1 | Do you have any trouble doing strenuous activities, like carrying a heavy shopping bag or a suitcase? | 1 | 2 | 3 | 4 |
| 2 | Do you have ang trouble taking a long walk? | 1 | 2 | 3 | 4 |
| 3 | Do you have ang trouble taking a short walk outside of the house? | 1 | 2 | 3 | 4 |
| 4 | Do you have to stay in a bed or a chair for most of the day? | 1 | 2 | 3 | 4 |
| 5 | Do you need help with eating, dressing, washing yourself or using the toilet? | 1 | 2 | 3 | 4 |
| 6 | Are you limited in any way in doing either you work or doing household jobs? | 1 | 2 | 3 | 4 |
| 7 | Are you limited in your hobbies or leisure activities? | 1 | 2 | 3 | 4 |
| DURING THE PAST WEEK: | |  |  |  |  |
| 8 | Were you short of breath? | 1 | 2 | 3 | 4 |
| 9 | Have you had pain? | 1 | 2 | 3 | 4 |
| 10 | Did you need to rest? | 1 | 2 | 3 | 4 |
| 11 | Have you had trouble sleeping? | 1 | 2 | 3 | 4 |
| 12 | Have you felt weak? | 1 | 2 | 3 | 4 |
| 13 | Have you lacked appetite? | 1 | 2 | 3 | 4 |
| 14 | Have you felt nauseated? | 1 | 2 | 3 | 4 |
| 15 | Have you vomited? | 1 | 2 | 3 | 4 |
| 16 | Have you been constipated? | 1 | 2 | 3 | 4 |
| 17 | Have you had diarrhea? | 1 | 2 | 3 | 4 |
| 18 | Were you tired? | 1 | 2 | 3 | 4 |
| 19 | Did pain interfere with your daily activities? | 1 | 2 | 3 | 4 |
| 20 | Have you had difficulty in concentrating on things, like reading a newspaper or watching television? | 1 | 2 | 3 | 4 |
| 21 | Did you feel tense? | 1 | 2 | 3 | 4 |
| 22 | Did you worry? | 1 | 2 | 3 | 4 |
| 23 | Did you feel irritable? | 1 | 2 | 3 | 4 |
| 24 | Did you feel depressed? | 1 | 2 | 3 | 4 |
| 25 | Have you had difficulty remembering things? | 1 | 2 | 3 | 4 |
| 26 | Has your physical condition or medical treatment interfered with your family life? | 1 | 2 | 3 | 4 |
| 27 | Has your physical condition or medical treatment interfered with your social activities? | 1 | 2 | 3 | 4 |
| 28 | Has your physical condition or medical treatment caused you financial difficulties? | 1 | 2 | 3 | 4 |
| FOR THE FOLLOWING QUESTIONS PLEASE CIRCLE THE NUMBER BETWEEN 1 AND 7 THAT BEST APPLIES TO YOU | | | | | |
| 29 | How would you rate your overall physical condition during the past week?  1 2 3 4 5 6 7  Very poor Excellent | | | | |
| 30 | How would you rate your overall quality of life during the past week?  1 2 3 4 5 6 7  Very poor Excellent | | | | |
